# Management of venous thromboembolism: clinical guidance from the Anticoagulation Forum

**DOI:** 10.1007/s11239-015-1320-5

**Published:** 2016-01-16

**Authors:** Jack E. Ansell

**Affiliations:** Hofstra North Shore-LIJ School of Medicine, Hempstead, NY USA

Venous thromboembolism (VTE), comprised of deep vein thrombosis (DVT) and pulmonary embolism (PE), has gained increasing recognition over the last two decades as a serious medical condition and a public health concern [1]. Since VTE is a largely preventable disorder, appropriately focused public health initiatives can have a major impact on its incidence. On average, about 1/1000 individuals will develop VTE annually, with the highest incidence in the older population [2]. Because VTE is a disease of aging we can only expect its incidence to rise with the graying of the population. Importantly, when VTE manifests itself as PE, it has a high case fatality rate and PE accounts for approximately 100,000 deaths per year [2]. Accurate statistics, however, are difficult to acquire, possibly because PE often develops as a complication of other serious conditions and it often goes undiagnosed, or even when diagnosed, death is attributed to the underlying illness.

What has changed about VTE that has elevated its importance in the spectrum of medical conditions? Many factors may be responsible, including a better understanding of the pathophysiology of VTE, a better recognition of risk factors leading to VTE, and a better understanding and recognition of hereditary and acquired thrombophilias. Undoubtedly, however, recent improvements in the treatment of VTE play an important role and make it more important than ever that healthcare providers are well educated about recent changes. We now have interventions such as thrombolysis, mechanical clot retrieval, new venous stents and vena cava filters or combinations of these [3], some of which have been shown to improve outcomes in those patients with the most severe forms of VTE. For the overwhelming majority of cases, however, treatment is based on the administration of an anticoagulant to prevent the extension or recurrence of thrombus [4]. Heparin was first used clinically in the 1930s followed shortly thereafter by dicoumarol and warfarin in the 1940s, and these agents have been the foundation of treatment of VTE until the present time [5]. For the first time since the discovery of the VKAs, we now have new oral anticoagulants that are alternatives to more traditional therapies in both the acute and long-term management of VTE [6]. These drugs induce anticoagulation by directly targeting and binding to specific coagulation factors and neutralizing them, a mechanism of action entirely different from the VKAs and one that has advantages as well as disadvantages. With warfarin, clinicians must be familiar with the pharmacokinetics of only one drug, yet management is complex and labor intensive and expert dose management is required to achieve the best outcomes [7]. The new direct oral anticoagulants (DOACs) are advantageous because they are easier to manage, but like so many other medication classes, there are now 4 such drugs on the market, each with their own indications, doses and pharmacokinetics [8]. Thus, the complexity of managing oral anticoagulant therapy persists, whether with the VKAs or the DOACs.

There are many therapeutic decision points in the course of VTE treatment. To aid clinicians, professional groups have published treatment guidelines based on the best medical evidence or rendered consensus opinions when good quality evidence is lacking. The most prominent of these guidelines are those published by the American College of Chest Physicians, starting in 1989 with updates every 3–4 years, the last being in 2012. With the rapidly changing management of VTE, in part, spurred by the introduction of the DOACs, the Anticoagulation Forum detected an urgent need for practical knowledge about the changing management paradigm. The Anticoagulation Forum is the largest peer organization of anticoagulation service providers in North America (ACForum.org). Its 6000+ members are comprised of physicians, nurses, and pharmacists representing over 2000 anticoagulation services (clinics). The AC Forum’s goal is to create a community where physicians, pharmacists and nurses can access the latest research, engage in educational programs, and find tools to evaluate and enhance their practice.

In an effort to bring practical and implementable information to its members and the provider community at large, the AC Forum initiated this guidance project by assembling experts in the disciplines of VTE research, education and care, asking them to consider practical everyday questions about the treatment of VTE using the best medical evidence and existing guidelines, but not hesitating to offer consensus opinions when high-quality evidence is not available. This special issue of the Journal of Thrombosis and Thrombolysis includes 12 manuscripts focused on the epidemiology and management of VTE with a particular emphasis on the management of drugs employed in its treatment, with both traditional agents as well as the new DOACs.
